# Comparison of Yang-Monti ileal ureter-bladder anastomosis versus Yang-Monti ileal ureter-ureteral anastomosis for the treatment of ureteral stenosis: a randomized controlled trial in a miniature pig model

**DOI:** 10.1186/s12894-019-0563-4

**Published:** 2019-12-10

**Authors:** Wang Zhenxing, Sun Zhaolin, Yang Xiushu, Luo Guangheng, Tian Ye, Shen Lei, Su Zhiyong, Liu Hongming

**Affiliations:** 1 Basic Medical College, Guizhou Medical University, Guiyang, 550004 China; 2 Department of Urology, Guizhou Provincial People’s Hospital affiliated to Guizhou Medical University, Guiyang, 550002 Guizhou China; 3 Department of Urology, Guizhou Provincial People’s Hospital, No. 83 East Zhongshan Road, Guiyang, 550002 Guizhou China; 4 Department of Surgical Operation Laboratory, Zunyi Medical University, No. 6 West Xuefu Road, Zunyi, Xinpu New District China; 5 Department of Urology, Guizhou Medical University, No. 9 Beijing Road, Guiyang, Guizhou China; 6 Department of Urology, Zunyi Medical University, No. 6 Xuefu West Road, Xinpu New District, Zunyi, Guizhou China

**Keywords:** Ureteral long stricture, Yang-Monti ileal ureter reconstruction, Ureter-bladder anastomosis, Ureter-ureteral anastomosis

## Abstract

**Background:**

The aim of the present study was to establish an animal model of Yang-Monti ileal ureter-bladder anastomosis and Yang-Monti ileal ureter-ureteral anastomosis and compare the advantages and disadvantages of the two surgical methods.

**Methods:**

Fourteen 12-month-old male Chinese miniature pigs weighing 21 ± 1.38 kg were randomly divided into two groups. Group A (*n* = 7) underwent end-to-end anastomosis of the left Yang-Monti ileal ureter, left ileal ureter and left lower ureter; group B (n = 7) underwent anastomosis of the left Yang-Monti ileal ureter, left ileal ureter and bladder. In both groups, the contralateral kidney was removed at 1 week postoperatively. The incision length and operation time of the two groups were compared. Changes in serum creatinine and urea nitrogen were observed preoperatively, and at 2, 6 and 12 weeks postoperatively. Venous pyelography and cystography were performed at 12 weeks postoperatively to determine the ureteral patency and vesicoureteral reflux. At 12 months postoperatively, urinary culture was performed, and the diameter and histological changes of the intestinal ureter were assessed.

**Results:**

Surgery was successfully completed in all 14 pigs. In group A, one pig died due to an anesthetic accident, and one pig died from a lung infection on postoperative day 4. In group B, one pig died from adhesive intestinal obstruction on postoperative day 7. The overall survival rate was 78.6%, and the 11 surviving pigs had no urinary or intestinal fistulae. Compared with group B, group A had a significantly longer surgical incision (30.86 ± 2.41 cm versus 26.71 ± 3.64 cm; *p* = 0.01) and shorter operation time (181.29 ± 15.10 min versus 157.71 ± 20.49 min; *p* = 0.02). The serum creatinine and urea nitrogen concentrations did not significantly differ between groups. All pigs had normal renal function pre- and postoperatively. There was no stenosis or obstruction on venous pyelography. The narrowest diameter of the ureter was significantly smaller in group B (5.90 ± 0.30 mm) than in group A (7.26 ± 1.06 mm; *p* = 0.01), but no contrast agent returned to the upper urinary tract in either group. *Escherichia coli* was detected on urine culture. In group A, one pig had obstruction of the ureteral ureter, while another had stenosis of the lower ureteral anastomosis. In group B, one pig had pelvic and intestinal ureteral dilatation; however, all anastomoses were patent. The ileal ureteral diameter was significantly larger in group A (9.40 ± 2.35 mm) than group B (6.62 ± 0.37 mm; *p* = 0.02). Two pigs in group A had separation of the transitional epithelium and columnar epithelial mucosa, with granulation tissue hyperplasia. The pigs with stenosis and obstruction had smooth fibrous tissue and smooth muscle of the anastomosis. In both groups, the two types of epithelial tissue were close together, and the intestinal villi were mildly atrophied and shortened.

**Conclusions:**

An animal model of Yang-Monti ileal ureter-bladder anastomosis was successfully established. Compared with Yang-Monti ileal ureter-ureteral anastomosis, Yang-Monti ileal ureter-bladder anastomosis is simpler, more reliable, and results in fewer complications.

## Background

With the widespread development of urological endoscopic surgery, iatrogenic injury has become one of the most common causes of ureteral injury [1], and the incidence of long-term ureteral injury is increasing. The most serious complication of ureteroscopy is ureteral avulsion, with an incidence of 0.06–0.45% [2–5]. Most cases of lower ureteral avulsion are treated with ureteral bladder replantation. However, multiple operations cause adhesions and stenosis in the lower ureter, poor blood supply to the ureter, difficulty in healing postoperatively, scarring, and result in a ureter with a small diameter and thin wall, which is not conducive to anastomosis.

The Yang-Monti technique for ileal ureteral reconstruction is used in clinical practice and has good curative effects [6–8]. The characteristics of this method are that the alternative ureteral tube diameter is the same as that of the original ureter, the intestinal tube is short, the diameter of the replacement tube is small, and the intestinal tube is saved. In addition, the absorptive and secretive functions of the intestinal segment used in ureteral reconstruction are decreased because the absorbent surface area is limited and the mucous production is decreased, preventing occlusion of the lumen and postoperative metabolic abnormalities.

It remains controversial whether there is a need for an anti-reflux procedure in ureteral reconstruction. Some studies report that there is no need to establish an anti-reflux anastomosis, while others advocate the establishment of anti-reflux procedures [9–11]; furthermore, there is reportedly no difference in efficacy in accordance with the performance of an anti-reflux procedure [12]. In the non-anti-reflux procedure, the ileal ureter was implanted into the bladder directly. The operation is relatively simple and convenient, the operation time is short, and the incision is small. However, there may be a backflow of urine from the bladder to the replacement ureter or ipsilateral kidney, resulting in postoperative intestinal dilatation, ascending infection, pyelonephritis, hydronephrosis, and impaired renal function [13]. To reduce the incidence of such complications, anti-reflux anastomosis involves the insertion of a nipple structure at the ileal bladder junction [14], a proximal ileal wall tunnel anti-reflux procedure [15, 16], and an extra-urinary ureter tunnel extension (Lich-Gregoir method) [7, 12].

When the lower ureter is well preserved, the ileal ureter can be anastomosed to the lower ureter, but this may cause postoperative complications such as mucus obstruction and anastomotic stenosis. If the lower ureter cannot be anastomosed, the intestinal ureter needs to be re-implanted in the bladder to prevent reflux. This is achieved via Yang-Monti ileal ureter and bladder anastomosis or Yang-Monti ileal ureter and lower ureteral anastomosis [7]. We established animal models of the Yang-Monti ileal ureter and bladder anastomosis and the Yang-Monti ileal ureter and lower ureteral anastomosis to evaluate which method is more convenient to perform, and to compare the advantages and disadvantages of both methods.

## Materials and methods

### Materials

Fourteen 12-month-old male experimental Guizhou miniature pigs weighing 21 ± 1.38 kg were obtained from the Experimental Animal Center of Zunyi Medical College. The pigs were randomly numbered and divided into two groups using a computer program. Group A (*n* = 7) underwent anastomosis of the left Yang-Monti ileal ureter and the left lower ureter; group B (*n* = 7) underwent anastomosis of the left Yang-Monti ileal ureter and bladder. The contralateral kidney was removed from both groups at 1 week postoperatively to establish the two experimental animal models. The experiment was approved by the Medical Ethics Committee of Guizhou Provincial People’s Hospital. All experimental procedures were conducted in accordance with local guidelines on the ethical use of animals and the Guide for the Care and Use of Laboratory Animals (National Institutes of Health, publication no. 85–23, revised 2011). Refinement refers to the improvement of conditions, the treatment of animals, and the improvement of animal welfare on the basis of scientific principles, or the improvement of experimental procedures and improvement of experimental techniques to avoid or alleviate the pain and nervousness of animals that are not related to the purpose of the experiment scientific method. The sample size was calculated based on a previous study [17] and the use of a formula. Although the present sample size was small, it was sufficient to identify statistically significant differences between the two groups.

### Surgical procedures and postoperative treatment

#### Surgical steps

##### Group A

**Fig. 1:**
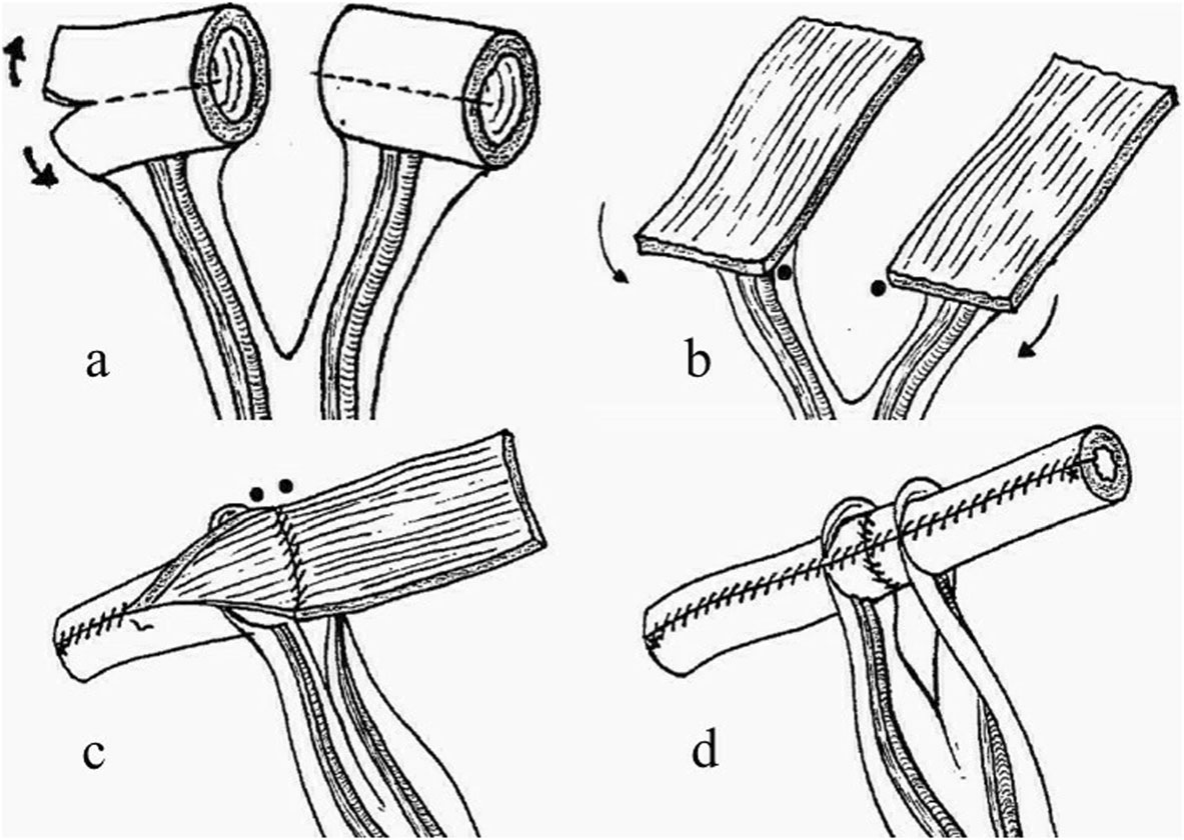
Photographs showing the method used to obtain the intestinal segments. This figure is from reference [17]. **a** Two 2.5-cm-long adjacent ileal segments are isolated and detubularized through longitudinal incisions on one side, 0.5 cm from the mesentery implantation, and (**b**) two identical pediculated flaps are obtained. **c**, **d** These flaps are attached by the two short branches and the resulting flap is tubularized; a tube with two long branches separated by two mesenteric insertions is obtained

**Fig. 2:**
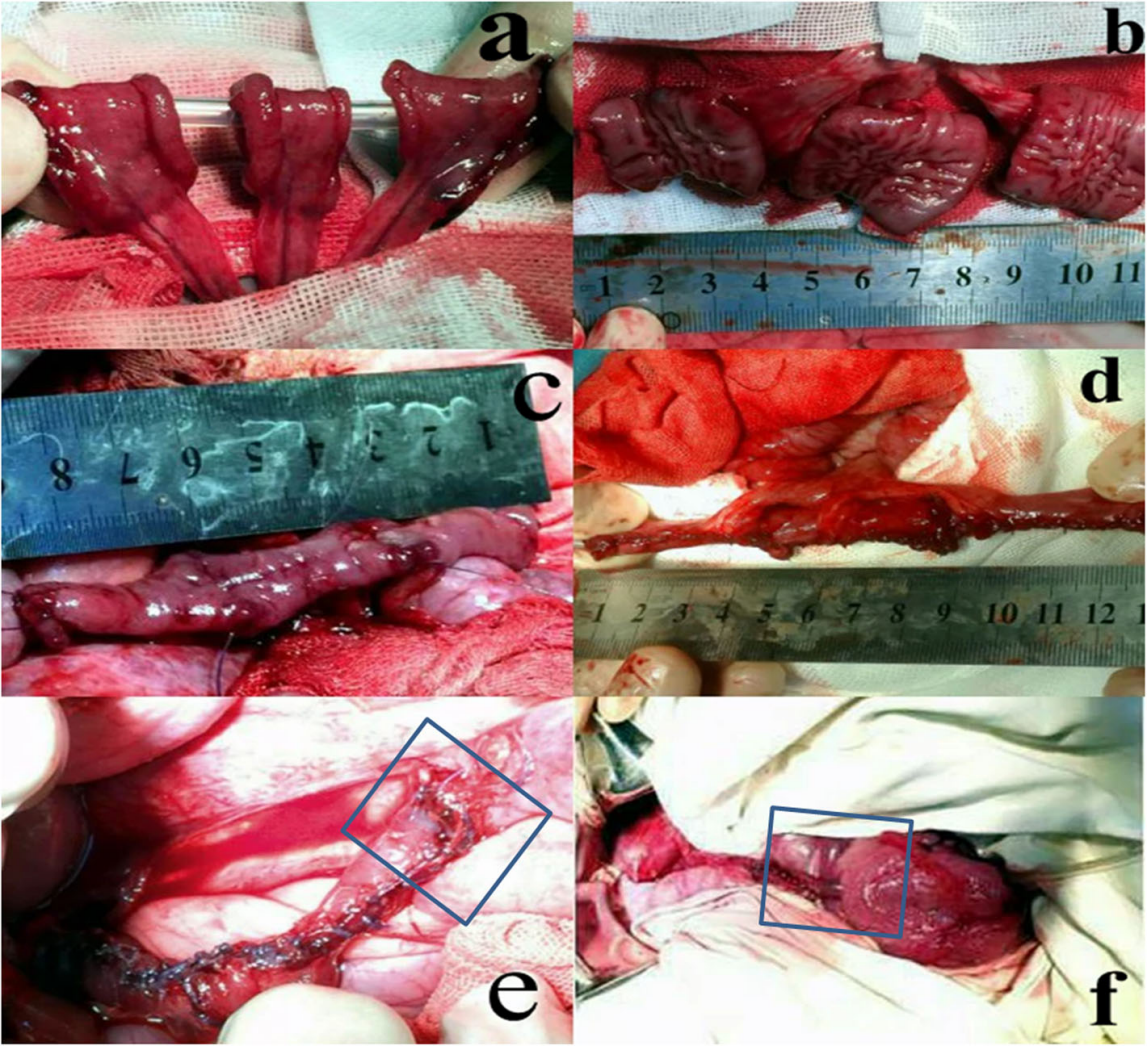
Photographs showing the preparation of the intestinal segments used as replacement ureters. **a** The selection of each 2-cm-long segment with independent and complete mesenteric vessels. **b** Longitudinal incision of three segments of the intestine to form a three-section rectangular intestine. **c** Sequential suture of the adjacent intestine; the pieces are connected into a long rectangular intestine. **d** The intestinal segment is sutured to form a long tubular structure. **e** The inferior ureter is anastomosed to the lower part of the ureter (the anastomosis is marked with a blue rectangular frame). **f** Anastomosis of the intestinal ureter and bladder (the anastomosis is marked with a blue rectangular frame)

1. Preoperative preparation: Animals were fasted for 24 h preoperatively and prevented from drinking water for 12 h preoperatively.
2. Anesthesia: After being weighed, anesthesia was induced via an intraperitoneal injection of 3% pentobarbital sodium (Shanghai Xinya Pharmaceutical Co., Ltd.) at a dose rate of 30 mg/kg and an intravenous infusion of propofol (Sichuan Guorui Pharmaceutical Co., Ltd.) at a dose of 1–2 mg/kg. Anesthesia was maintained with an intravenous infusion of propofol at a dose of 1–2 mg/kg/h.
3. Abdominal skin preparation: The skin was disinfected with 2.5% iodophor and the surgical area was draped with a sterile surgical towel. The skin was incised over the left rectus abdominis, and the subcutaneous tissue, muscle, and peritoneum were incised.
4. Creation of a model of extensive ureteral injury: The left side of the colon was moved medially to reveal the left posterior peritoneum. The left posterior peritoneum was incised to enable the identification of the left ureter. The left ureter was cut at the renal pelvis, the lower ureter was preserved,. The middle and upper segments of the ureter were removed to create a model that replicated actual extensive ureteral injury.
5. Selection of the intestinal segments: At a distance of 40 cm from the ileocecal junction, an ileal segment with an independent mesenteric blood supply was removed, and the surrounding tissues were protected with gauze.
6. Restoration of intestinal continuity: The two cut ends of the intestine were thoroughly washed with physiological saline before being disinfected with 2.5% iodophor. The intestine was then anastomosed with 5–0 non-absorbable suture. The whole-layer was sutured first, and then the muscular layer was sutured to restore the continuity of the intestine and close the mesangial hole.
7. Preparation of the intestinal ureter: The intestine was cut using a previously described method [17] (Fig. 1). The intestine was thoroughly cleaned and disinfected with saline and 2.5% iodophor before being cut into three 2-cm-long segments with independent and intact mesenteric vessels (Fig. 2a). A cross-section of the intestine was taken from the mesentery at the 6 o’clock position. The three intestinal segments were cut longitudinally (one was cut at the 9 o’clock position, one at the 12 o’clock position, and one at the 3 o’clock position) to create three rectangular sections of intestine (each of which was 4–6 cm long and 1.5 cm wide) (Fig. 2b). The adjacent intestinal pieces were sequentially sutured with an antibacterial micro-chord line (4–0) to form one rectangular piece of intestine with a length of 12–18 cm and a width of 2 cm (Fig. 2c). The piece of intestine was wrapped around a 30-cm-long F12 silica ureteral stent tube and sutured longitudinally with 5–0 absorbable suture (Johnson & Johnson) to form a long tubular structure to serve as a ureteral replacement segment (Fig. 2d).
8. Reconstruction of the ureter and ureteral stump anastomosis: One end of the ureteral stent was placed in the renal pelvis, while the other end of the ureteral stent was inserted into the bladder through the native ureteral stump. A minimal incision was made in the apex of the bladder, and the stent tube was pulled out of the bladder to create a cystostomy. The bladder incision was sutured. The upper end of the reconstructed ureter was anastomosed to the native ureteral stump with 5–0 absorbable suture. The lower end of the reconstructed ureter was anastomosed to the remaining lower end of the native ureter (Fig. 2e).
9. The distal end of the ureteral stent tube was placed under the skin of the left lower abdomen, and the incision was closed in a layered fashion.

##### Group B

Steps (1)–(7) and (9) were the same as for group A.

(8) Anastomosis of the reconstructed ureter and the bladder stump: One end of the ureteral stent tube was placed in the renal pelvis, while the other end was placed in the bladder on the left side. A minimal incision was made in the apex of the bladder, and the stent tube was pulled out of the bladder to create a cystostomy. The bladder incision was sutured. The upper end of the reconstructed ureter was anastomosed to the native ureteral stump with 5–0 absorbable suture. The lower end of the reconstructed ureter was directly anastomosed to the bladder (Fig. 2f).

In both groups, the contralateral kidney and ureter were removed at 1 week postoperatively.

#### Postoperative treatment

All pigs were fasted for 24 h postoperatively before beginning a liquid diet on postoperative day 2. Normal feeding was recommenced 3–5 days after the initiation of the liquid diet. Pigs were administered a daily intramuscular injection of penicillin (1.5 ml/kg) and metronidazole (50 ml/day) for 5 days postoperatively. The ureteral stent tube was left in place for 4 weeks before being surgically removed. The pigs were anesthetized(3% pentobarbital sodium, 30 mg/kg) via intraperitoneal injection before the collection of tissues and organs for examination. After the specimens were collected, the pigs were euthanized via an intravenous injection of 3% pentobarbital sodium (100 mg/kg).

### Observation indicators and data collection

The following data were collected for all pigs in both groups:

1. Operation time and length of the surgical incision.
2. Blood samples were taken to evaluate the serum creatinine and urea nitrogen concentrations preoperatively and at 2, 6, and 12 weeks postoperatively.
3. Intravenous pyelography and cystography were performed at 12 weeks postoperatively to observe the ureteral patency and vesicoureteral reflux.
4. Urine was collected from the bladder at 12 months postoperatively and cultured to check for the presence of urinary tract infection.
5. Gross macroscopic observation and histological examination of the intestinal ureter were performed at 12 months postoperatively to evaluate the pathological changes of the anastomosis and intestinal ureter.

### Statistical analysis

Animal grouping was designed in accordance with the principles of equilibrium, control, and repetition. All measurement data were expressed as the mean ± standard deviation. The *t-*test was used to compare data between the two groups. Differences were considered statistically significant at *p* < 0.05. Statistical analysis was performed using SPSS 24.0 software.

## Results

**Fig. 3:**
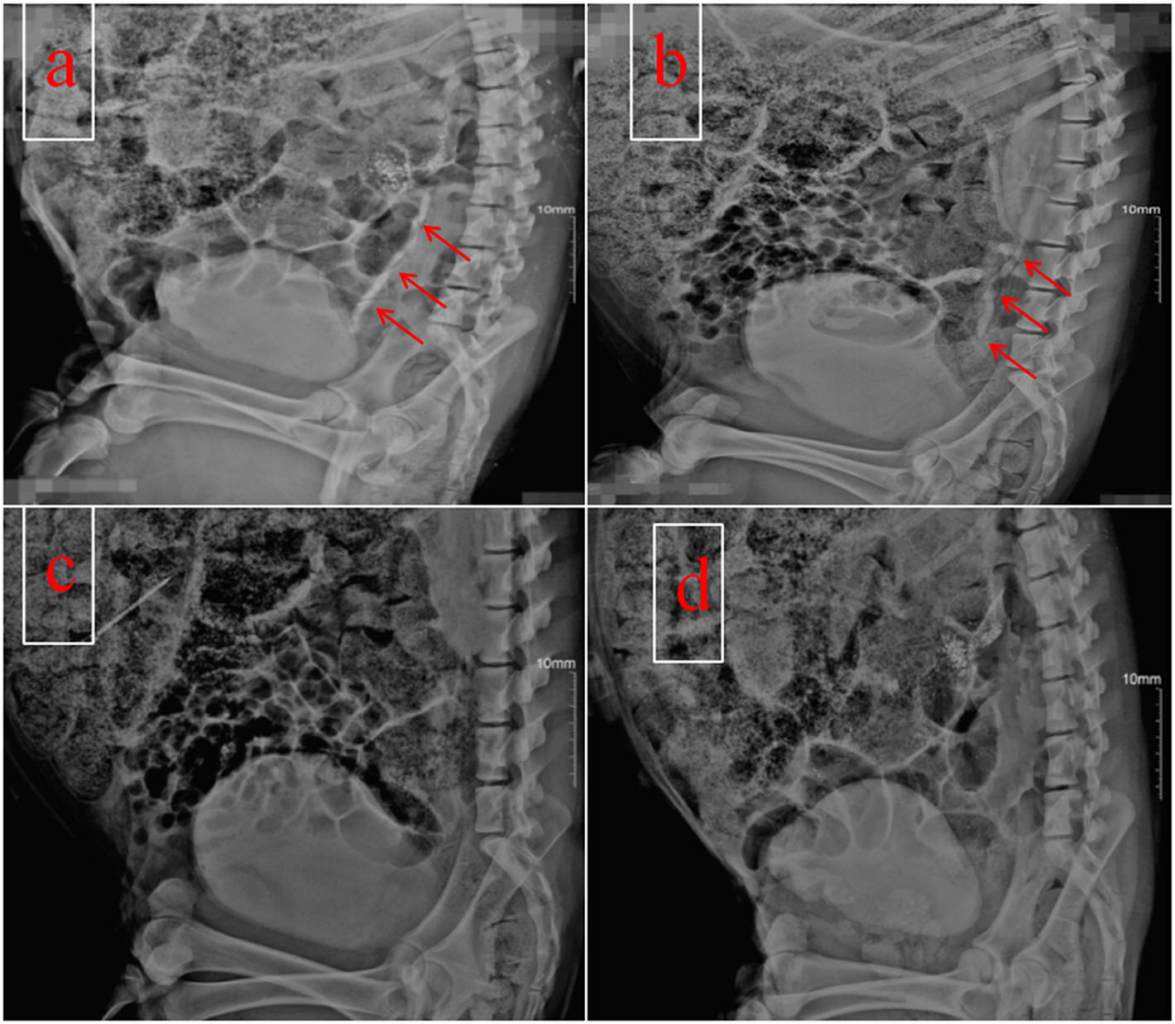
Intravenous pyelography and cystography findings at 12 weeks postoperatively. **a** Intravenous pyelography of a pig in group B. **b** Intravenous pyelography of a pig in group A. The red arrow indicates the lack of stenosis and obstruction in both groups. **c** Cystography of a pig in group A. **d** Cystography of a pig in group B. There is no reflux of the contrast agent to the upper urinary tract

The operation was successfully completed in all 14 miniature pigs. In group A, one pig died from an anesthetic accident, and another died from a lung infection on postoperative day 4. In group B, one pig died from adhesive intestinal obstruction on postoperative day 7. The overall survival rate was 78.6%, and the 11 surviving pigs had no urinary or intestinal fistulae. Compared with group B, group A had a significantly longer operation time (*p* = 0.01) and skin incision length (*p* = 0.02) (Table 1). The serum creatinine and urea nitrogen concentrations did not significantly differ between groups at any timepoint (*p* > 0.05). All pigs had normal renal function pre- and postoperatively (Table 2). Intravenous pyelography showed no stenosis or obstruction (Fig. 3a, b). The narrowest diameter of the ureter in group A was significantly larger than that in group B (*p* = 0.01) (Table 3). No contrast agent returned to the upper urinary tract in either group (Fig. 3c, d). *Escherichia coli* was detected in postoperative urine cultures from four pigs in group A and four pigs from group B.

**Table 1:** Length of surgical incision and operation time $\left(\overline{x}\pm s\right)$

Groups	Numbers of samples	Surgical incision length (cm)	operation time (min)
Group A	7	30.86 ± 2.41	181.29 ± 15.10
Group B	7	26.71 ± 3.64	157.71 ± 20.49
*P* Value		0.01	0.02

**Table 2:** Serum creatinine and urea nitrogen values of each group before and after surgery $\left(\overline{x}\pm s\right)$

Groups	Time	Sample number	Cr (μmol/L)	BUN (mmol/L)
Group A				
	Before surgery	5	102.30 ± 27.60	5.67 ± 1.34
	2 weeks after surgery	5	113.80 ± 14.22	7.35 ± 3.63
	6 weeks after surgery	5	117.70 ± 30.09	6.01 ± 1.37
	12 weeks after surgery	5	102.00 ± 3.89	5.69 ± 0.91
Group B				
	Preoperative	6	101.10 ± 22.18	5.97 ± 1.07
	2 weeks after surgery	6	101.10 ± 5.53	6.24 ± 1.08
	6 weeks after surgery	6	102.10 ± 3.99	6.11 ± 1.08
	12 weeks after surgery	6	100.70 ± 15.19	6.35 ± 1.20

**Table 3:** Intestinal ureteral diameter $\left(\overline{x}\pm s\right)$

Groups	Number of samples	Intestinal ureteral diameter (mm)	
		Intravenous	pyelography
Group A	5	7.26 ± 1.06	9.40 ± 2.35
Group B	6	5.90 ± 0.30	6.62 ± 0.37
*P* value		0.01	0.02

**Fig. 4:**
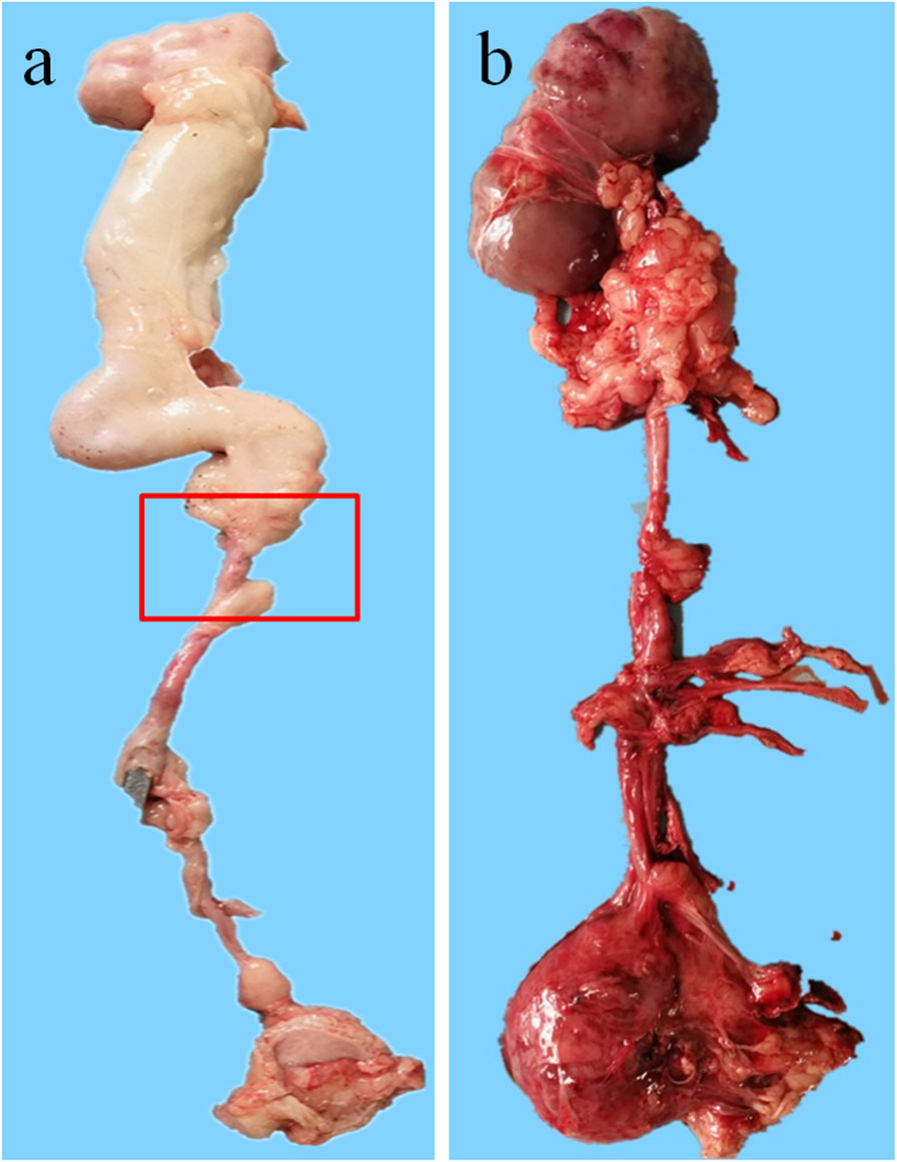
Photographs showing the gross examination of the intestinal ureters collected at 12 months postoperatively. **a** Stenosis of the inferior ureter and the distal end of the ureter in a pig in group A. The stenosis is marked with a red rectangular frame. There is obvious expansion of the upper ureter and hydronephrosis; the renal parenchyma is thin, and the kidney is small. **b** In a pig from group B, the ileal ureter and anastomosis are unobstructed, with no hydronephrosis

Gross examination of the collected specimens revealed complete obstruction of the distal ends of the inferior and lower ureters and narrowing of the other ends of the inferior and lower ureters in two pigs from group A. There was marked adhesion around the two anastomoses and obstructive stenosis distal to the intestinal ureter. The kidneys were atrophic with marked hydronephrosis and thin and transparent renal parenchyma (Fig. 4a).

In group B, one pig had moderate hydronephrosis and mild dilation of the intestinal ureter at 12 months postoperatively; however, the ileal ureter and anastomoses were patent (Fig. 4b). The rest of the pigs in group B had patent intestinal ureters and smooth anastomoses. The intestinal ureters were a reddish color, with a shape that was similar to that of the normal ureter. The intestinal ureters were slightly thicker than the normal ureters, and were surrounded by fibrous tissue and adipose tissue. There was continuous, intestinal mucosal atrophy on the surface of the intestinal ureters, with no signs of hydronephrosis.

The ileal ureteral diameter was significantly larger in group A than group B (*p* = 0.02) (Table 3).

**Fig. 5:**
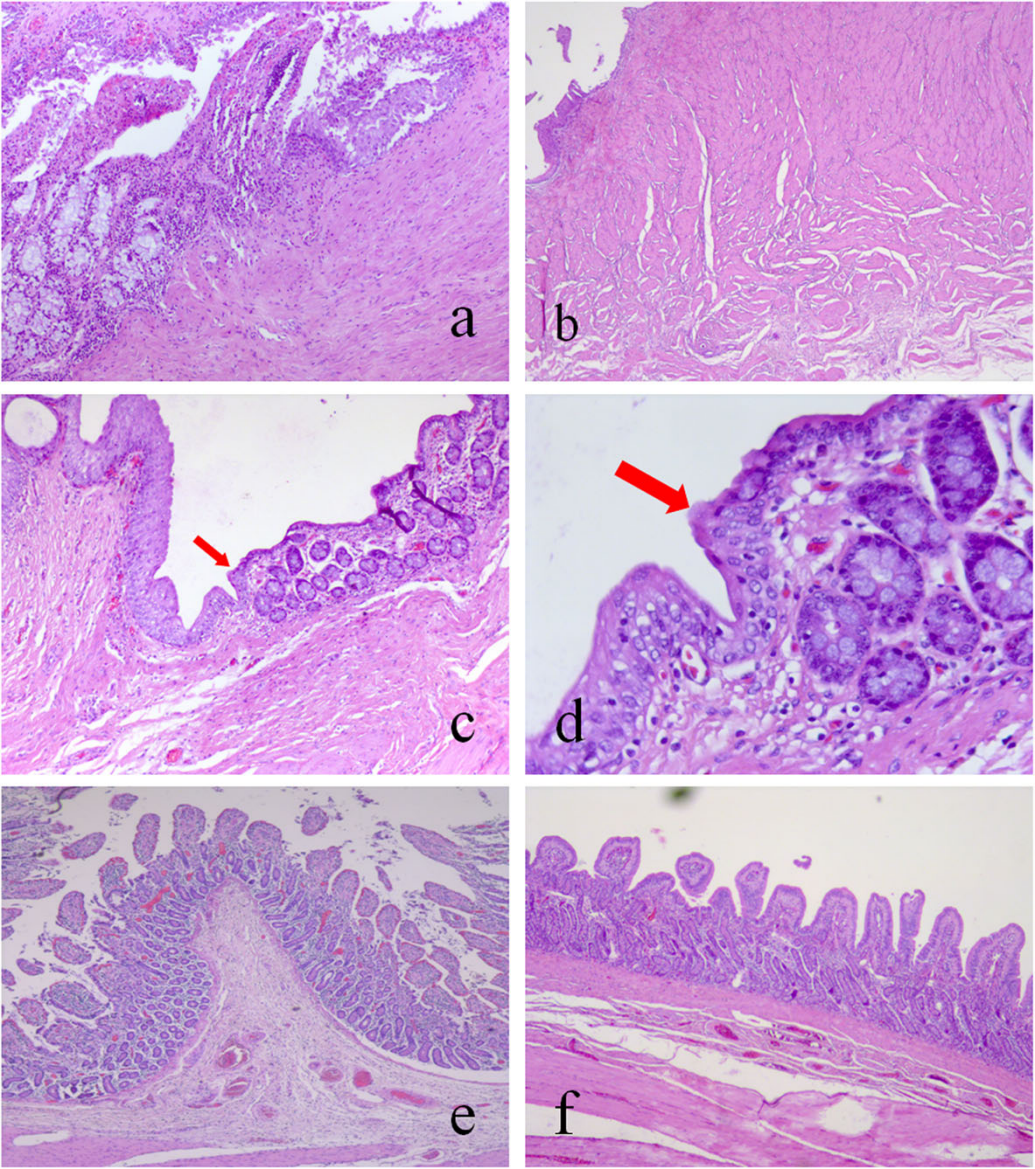
Histological examination of the intestinal ureters at 12 months postoperatively. **a** In group A, the transitional epithelium and columnar epithelial mucosa are not close together, there is hyperplasia of the granulation tissue, lamina propria, and fibrous tissue (Hematoxylin-eosin stain (H-E); 100× magnification). **b** In group B, there is hyperplasia of the lamina propria, myometrial fibrous tissue, and smooth muscle, with hyaline degeneration (H-E; 40× magnification). **c** On the left side of the transitional epithelium and the right side of the intestinal epithelium,the two types of epithelial tissue were close together (H-E; 100× magnification). **d** On the left side of the transitional epithelium and the right side of the intestinal epithelium, there are transitional epithelial cells (marked with red arrows) crawling toward the intestinal mucosa and covering part of the intestinal mucosa (H-E; 400× magnification). **e** Normal intestinal villi (H-E; 40× magnification). **f** Ileal ureteral villi (H-E; 40× magnification)

Histological examination showed that two pigs in group A had separation between the transitional epithelium of the upper and lower ends of the ureters and the columnar epithelium, with granulation tissue hyperplasia. Two pigs in group A with ureteral obstruction had fibrous tissue and smooth muscle tissue hyperplasia and support stenosis at the anastomosis site (Fig. 5a, b). In the six surviving pigs in group B, the two types of epithelial tissue were close together in the ureteral ureters at both ends of the anastomosis (Fig. 5c, d). In both groups, the replacement intestinal segments had shortened and atrophied villi compared with normal intestinal villi (Fig. 5e, f).

## Discussion

In the present study, we successfully established an animal model of ileal ureter-bladder anastomosis and ileal ureter-ureteral anastomosis using the Yang-Monti technique. The serum creatinine and urea nitrogen concentrations did not significantly differ between the two groups at 2, 6, and 12 weeks postoperatively. The renal function was stable, with no postoperative renal dysfunction or failure.

The average operation time and length of the surgical incision in group A were significantly longer than in group B. This may be because the operational procedures in group B were relatively easier to perform than those in group A. In group A, the anastomosis between the intestinal ureter and the native ureter required the lower ureter to be fully freed. As the lower ureter is deeply located, the operational space available for the anastomosis was relatively small. In contrast, the intestinal ureter in group B was directly anastomosed to the bladder, and the lower segment of the ureter was not required. The bladder of the miniature pig is more easily recognized when it is full, and the apex wall, the two lateral walls, and the anterior and posterior walls of the bladder are not connected to the surrounding pelvic wall. As the position of the bladder in the pelvic cavity is not fixed, the bladder is movable and easier to find than the lower part of the ureter. Furthermore, it is easy to re-integrate the ureter and the left side of the bladder, and the operational space is large.

The most common complication of ileal ureteral surgery is urinary tract infection, which is related to bladder ileal reflux and intestinal mucus secretion; this complication is difficult to control and is often recurrent, eventually leading to hydronephrosis or multiple renal cortex abscesses and pyelonephritis [7, 18, 19]. Urine culture results show that the most frequently cultured organism is *E. coli*, followed by *Klebsiella pneumoniae* [12]. Yao et al. [20] performed short- and long-term observations of 60 patients with ileal ureters, and found abnormal changes in the urine (including a small amount of protein, white blood cells, pus balls, and intestinal mucus); however, only four patients had clinical symptoms of urinary tract infection, and the symptoms disappeared after treatment with antibiotics. Therefore, urinary changes do not necessarily indicate the presence of urinary tract infection, and antibiotic treatment is needed only when symptoms of acute infection occur [20]. The *E. coli* colonization seen in urine cultures in the present study is consistent with the literature. *E. coli* is a pathogenic pathogen of the intestine, and may move from the intestine to the urinary tract during surgery; no antibiotic treatment is required for such asymptomatic bacterial urinary infection.

The two methods used in ileal ureteral surgery and bladder anastomosis are non-anti-reflux and anti-reflux. In the non-anti-reflux procedure, the ileal ureter replaces the ureter directly after the bladder. The operation is relatively simple, the operation time is short, and the incision is small. However, there may be postoperative intravesical urine reflux to the replacement ureter or ipsilateral kidney, resulting in postoperative intestinal dilatation, ascending infection, pyelonephritis, hydronephrosis, and impaired renal function [13]. The incidences of such complications are reduced by the performance of anti-reflux anastomosis. In the present study, there was no stenosis obstruction observed in either group. However, the narrowest diameter of the intestinal ureter was significantly larger in group A than in group B.

On intraoperative measurement, the reconstructed intestinal ureteral diameter was still larger than the normal ureteral tube diameter. In group A, the ureter was cut longitudinally before the intestinal ureter and the lower ureter were anastomosed. Thus, the anastomosis is relatively small, the ileum is more stretched than the ureter, and the urine flows through the relatively narrow lower ureter after passing through the wide intestinal ureter. The urine flow rate is slowed, the stagnation time is long, and urine is retained in the intestinal ureter, which causes the intestinal ureter to expand; the indwelling endoscopic tube also causes postoperative expansion. In contrast, the intestinal ureter in group B was directly matched with the bladder, and the anastomosis was wider than that in group A. Venous pyelography showed that the contrast agent quickly entered the bladder through the intestinal ureter without being retained. This may be why the intestinal ureteral diameter in group B was smaller than that in group A.

As the Yang-Monti ileal ureter is directly matched with the bladder, the anastomosis is large, which may increase the likelihood of reflux. In the present study, no contrast agent returned to the upper urinary tract in either group. In group A, there was no reflux because the intestinal ureter and the lower ureter were anastomosed and the anti-reflux mechanism between the ureter and the bladder was retained. There was also no reflux in group B, even though the anastomosis of the intestinal ureter was large and directly opened to the bladder. The absence of reflux in group B may be because the intestinal ureteral diameter was close to that of the normal ureter, and the intestinal ureter had good elasticity and a long length. The ileum retains its peristaltic function after replacing the ureter, which is beneficial in preventing intravesical pressure [7, 12]. However, confirmation of this requires measurement of the intestinal ureter, renal pelvis, and intravesical pressure. Other possible reasons for the absence of reflux in group B are that the miniature pig has a short urethra, good bladder elasticity, and rapid urine emptying. Therefore, there is little need to establish an anti-reflux mechanism in the Yang-Monti ileal ureter in miniature pigs.

The intestinal epithelium is a single-layer columnar epithelium, while the urinary tract is a transitional epithelium. The examination of the intestinal tract after ileal ureter replacement showed that all of the anastomotic regions had unobstructed lumens, no epithelial proliferative changes in the junctional zone, and were located less than 1 cm from the adjacent junctional zone; this very short distance is covered by migration epithelial metaplasia [21]. In a previous study of ileal ureter reconstruction via the Yang-Monti method in rabbits, histological sections obtained 12 weeks postoperatively showed that stratified transitional epithelial cells had spread toward and covered part of the intestinal mucosa at the site of the anastomosis, and the intestinal mucosa on the inner surface of the ureter was obviously atrophied [22]. In another study in which ileal ureteral surgery was performed on miniature pigs, the columnar epithelium was still visible in the middle ileum at 3 years postoperatively; however, the villi were atrophied and some of the villi had become shorter and wider than normal [23]. In the present study, histological examination of the intestine and ureter was consistent with the findings of these previous studies. After the ileum was used to replace the ureter, its histological characteristics changed to adapt to the urinary environment. The transitional epithelium and the columnar epithelium were continuous and replaced the segmental columnar epithelium, indicating that the ileum that replaced the ureter maintained the shape and integrity of the epithelium, which may still have a mucosal barrier. Further experimental studies are needed to confirm whether the intestinal mucosal barrier changes in the urinary environment. In addition, after the ileum was used to replace the ureter, the intestinal mucosa shrank, the villi became shorter, and the absorption and secretion functions of the intestinal mucosa were correspondingly weakened. This may be related to the absence of obvious metabolic abnormalities and mucus secretion after Yang-Monti ileal ureteral reconstruction in the clinical setting, but requires confirmation in further studies of the absorption and secretion functions of intestinal mucosal epithelial cells.

In group A, the distal end of the inferior ureter and the lower end of the ureter were completely obstructed and stenotic, and there was marked hydronephrosis. In group B, one pig had hydronephrosis and mild dilation of the intestinal ureter, although the anastomosis was smooth. Histological examination showed that group A had hyperplasia of fibrous tissue and smooth muscle at the anastomosis site, suggesting the presence of stenosis or obstruction. In group A, the intestinal ureter was anastomosed with the lower ureter, which means that the lower ureter needed to be separated from the surrounding tissue. The thin ureteral wall may lead to poor blood supply in the lower ureter; thus, although there is good blood supply to the ileal ureter, there may be poor blood supply between the intestinal ureter and the lower ureter after anastomosis, resulting in fibrosis or scarring postoperatively. This may be why the incidence of stenosis or obstruction and hydronephrosis was higher in group A than in group B. In group B, the intestinal ureter was directly matched with the rich blood supply of the bladder, which limited the occurrence of postoperative stenosis or obstruction. Although there was no evidence of hydronephrosis and regurgitation on intravenous pyelography and cystography in group B, visual observation suggested the presence of hydronephrosis and mild dilation of the intestinal ureter. Furthermore, although the short-term imaging examination revealed no abnormal changes, the inguinal ureter may undergo compensatory changes over time after countering the persistent intravesical pressure.

## Conclusions

We successfully established an animal model of Yang-Monti ileal ureteral reconstruction. Compared with the Yang-Monti ileal ureter-ureteral anastomosis, the Yang-Monti ileal ureter-bladder anastomosis is simpler, more reliable, and results in fewer complications.

## Data Availability

The datasets used and/or analysed during the current study are available from the corresponding author on reasonable request.
